# Self-Reported Prevalence and Predictors of HIV and Gonorrhea Among Primary Healthcare Attendees: A Cross-Sectional Study from Saudi Arabia

**DOI:** 10.3390/healthcare14101369

**Published:** 2026-05-16

**Authors:** Saad Alshahrani, Badr F. Al-Khateeb, Roa Altaweli, Raed Aldahash, Noof Alwatban, Maryam Alhabas, Wejdan Ali AlNowaisir, Amani Alharthy, Lubna Alnaim, Abeer Almudaihim, Ashraf El-Metwally

**Affiliations:** 1 Department of Surgery, College of Medicine, Prince Sattam bin Abdulaziz University, Alkharj 16273, Saudi Arabia; s.alshahrani@psau.edu.sa; 2 College of Medicine, King Saud bin Abdulaziz University for Health Sciences, Riyadh 14611, Saudi Arabia; khateebb@mngha.med.sa (B.F.A.-K.); dr.raaed@hotmail.com (R.A.); 3 Ministry of the National Guard-Health Affairs, Riyadh 11426, Saudi Arabia; watbann@ksau-hs.edu.sa (N.A.); alhabasma@ksau-hs.edu.sa (M.A.); nuaimlu@ksau-hs.edu.sa (L.A.); mudaiheema@ksau-hs.edu.sa (A.A.); 4 King Abdullah International Medical Research Center, Riyadh 11481, Saudi Arabia; 5 Department of Maternity and Pediatric Nursing, College of Nursing, Princess Nourah bint Abdulrahman University, Riyadh 11671, Saudi Arabia; rfaltaweli@pnu.edu.sa; 6 College of Public Health & Health Informatics, King Saud bin Abdulaziz University for Health Sciences, Riyadh 11481, Saudi Arabia; 7 College of Public Health, University of Imam Abdulrahman bin Faisal, Dammam 34212, Saudi Arabia; wjdan3ali@gmail.com; 8 Department of Health Science, College of Health and Rehabilitation Sciences, Princess Nourah bint Abdulrahman University, Riyadh 11671, Saudi Arabia; afalharthy@pnu.edu.sa; 9 College of Applied Medical Sciences, King Saud bin Abdulaziz University for Health Sciences, Al-Ahsa 31982, Saudi Arabia; 10 College of Applied Health Sciences, King Saud bin Abdulaziz University for Health Sciences, Riyadh 11481, Saudi Arabia

**Keywords:** HIV, gonorrhea, sexually transmitted infections, self-reported diagnosis, prevalence, predictors

## Abstract

**Background/Objectives:** This study aimed to estimate self-reported prevalence of HIV and gonorrhea among primary healthcare attendees in Riyadh and to identify key demographic, behavioral, and clinical predictors, acknowledging that diagnoses were based on participant self-report rather than laboratory confirmation. **Methods**: A cross-sectional survey was conducted between March and July 2023 across 48 primary healthcare centers in Riyadh. A total of 14,239 adult participants (aged ≥18 years) completed an electronically administered questionnaire that included self-reported prior diagnoses of HIV and gonorrhea. Multivariable logistic regression models were used to identify independent predictors of self-reported HIV and gonorrhea. **Results**: The self-reported prevalence of HIV was 2.6% (95% CI: 2.35–2.87%), and gonorrhea was 3.1% (95% CI: 2.83–3.40%). Several factors were independently associated with higher odds of self-reported infection. Younger age (<50 years) increased risk (HIV: AOR = 2.19; gonorrhea: AOR = 1.57), as did female sex (HIV: AOR = 1.67; gonorrhea: AOR = 1.59), higher education (HIV: AOR = 1.29; gonorrhea: AOR = 1.23), married status (HIV: AOR = 1.76; gonorrhea: AOR = 1.49), and insurance coverage (HIV: AOR = 2.01; gonorrhea: AOR = 1.88). Behavioral and clinical factors included smoking (HIV: AOR = 4.79; gonorrhea: AOR = 2.41), hypertension (HIV: AOR = 2.58; gonorrhea: AOR = 1.49), obesity (HIV: AOR = 11.55; gonorrhea: AOR = 9.02), hypercholesterolemia (HIV: AOR = 2.24; gonorrhea: AOR = 2.53), and heart disease (HIV: AOR = 11.31; gonorrhea: AOR = 8.77). The notably high associations for obesity and heart disease should be interpreted with caution, as they may be influenced by detection bias or residual confounding within the healthcare-seeking sample. **Conclusions**: This study provides key insights into the self-reported burden and predictors of HIV and gonorrhea in Saudi Arabia. While identifying significant demographic and metabolic risk profiles, the high magnitude of certain clinical associations must be interpreted with caution due to potential detection bias and residual confounding. Given the reliance on self-reported data, these findings should be viewed as an epidemiological baseline rather than absolute prevalence. Prioritizing clinical context over statistical values and strengthening integrated, laboratory-based surveillance within primary care will be essential for improving early detection and evidence-based prevention strategies in the region.

## 1. Introduction

Sexually transmitted infections (STIs), particularly human immunodeficiency virus (HIV) and gonorrhea, remain a critical global public health concern due to their association with chronic complications and social stigma [1,2]. While global prevalence is well-documented, epidemiology varies by region and is heavily influenced by sociocultural contexts and healthcare access [3,4,5,6,7]. Notably, gonorrhea rates have surged in several high-income countries, and STDs continue to disproportionately affect low- and middle-income countries, where young people and individuals with limited healthcare access are particularly vulnerable [8,9,10,11,12]. In the Middle East and North Africa (MENA), the STI burden is likely underrecognized due to limited surveillance and reporting barriers [13,14]. Longitudinal data show that the absolute number of incident cases in the MENA region doubled between 1990 and 2021, reaching 55.8 million, driven by population growth and gaps in early detection [15].

In Saudi Arabia, the epidemiological landscape is less defined due to sociocultural sensitivities and a reliance on passive reporting [16,17,18]. Although conservative norms might suggest a lower prevalence, underreporting and limited routine screening may mask the actual burden [19,20,21]. Notably, the country’s unique demographic profile—characterized by rapid urbanization and a substantial expatriate workforce—introduces complex dynamics to STI transmission. Increased international mobility and the influx of individuals from diverse epidemiological backgrounds may influence local disease distribution and risk patterns [14,15]. Furthermore, evolving lifestyle behaviors among the growing young adult population further necessitate a re-evaluation of current surveillance strategies. Most existing local studies are limited by small sample sizes or outdated datasets, restricting their utility for modern public health planning [20,21,22].

The primary objective of this study was to estimate the self-reported prevalence of HIV and gonorrhea among adults attending primary healthcare centers in Riyadh. Secondarily, the study aimed to identify demographic, behavioral, and clinical predictors associated with these infections. We hypothesized that younger age and established behavioral factors would be expected predictors of higher infection odds, while metabolic comorbidities were included as exploratory predictors to investigate their potential association within the Saudi healthcare context, acknowledging that increased healthcare contact might influence diagnosis rates. By focusing on a large and diverse primary healthcare population, this study provides important epidemiological evidence to improve understanding of STI burden and risk factors within the Saudi Arabian healthcare context.

## 2. Materials and Methods

### 2.1. Study Design, Setting, and Sampling Framework

This study employed a cross-sectional design conducted over a five-month period, from March to July 2023. The research was part of a national initiative launched in 2021 to restructure Saudi Arabian healthcare into a health cluster model, aimed at enhancing service efficiency and integration. We focused on the Riyadh region, which is administratively divided into three health clusters overseen by the Central Health Services authority. Specifically, we concentrated on Health Cluster 2. This network serves an estimated 3.7 million individuals through 105 primary healthcare centers (PHCs) alongside secondary and tertiary facilities. We selected this cluster for its demographic diversity and robust infrastructure, which represent both urban and suburban populations. To ensure balanced geographic representation, we utilized a stratified random sampling approach to select 48 PHCs. Stratification was based on center location (urban vs. suburban), allowing for the proportional inclusion of different community settings and enhancing the generalizability of our findings.

### 2.2. Sampling Strategy and Participant Selection

To ensure representativeness, a probabilistic, multi-stage stratified random sampling design was implemented. In the first stage, 48 PHCs were randomly selected from the 105 centers within Health Cluster 2. We utilized stratified random sampling based on geographic location (urban vs. suburban) to ensure proportional representation across different population settings [23]. This approach ensured that each PHC had a known, non-zero probability of selection. In the second stage, we recruited participants within each selected center using systematic random sampling. Specifically, a sampling interval (k) was calculated by dividing the estimated daily patient volume by the target number of participants required per center [23]. A random starting point was selected each day, and every kth eligible individual was invited to participate. This approach ensured a structured probabilistic sampling process and minimized reliance on convenience sampling.

The target sample size was calculated using established epidemiological standards for estimating population proportions. To ensure maximum variance, we assumed a conservative prevalence estimate with a 95% confidence level and a strict 1% margin of error. These parameters determined a minimum required sample size of 9604 participants. To account for potential non-response or incomplete submissions, we adjusted the recruitment target upward. Our final sample of 14,239 participants significantly exceeds the initial requirements. This sample size provides robust statistical power to detect low-prevalence outcomes and ensures high stability for the multivariable regression models.

### 2.3. Inclusion and Exclusion Criteria

The study population comprised adults aged 18 years and older who attended the selected PHCs, including both Saudi citizens and non-Saudi residents. Participants were eligible if they were seeking care or accompanying patients during the study period and could independently complete the survey. We excluded individuals who met any of the following criteria: employment as a staff member or healthcare worker at the participating PHCs, being under age 18, or having cognitive conditions that could interfere with survey comprehension. Additionally, those who refused informed consent were excluded. To prevent duplicate entries, we strictly excluded repeat attendees. Unique participant identifiers and digital metadata, such as device IDs, ensured that each individual provided only a single response. Following the application of these criteria, 14,239 respondents were included in the final dataset for analysis.

### 2.4. Survey Instrument and Data Collection Procedures

Data were gathered using a digitally administered questionnaire, originally developed in English based on validated international sources. To ensure linguistic and conceptual accuracy, the tool underwent a forward translation into Arabic followed by a back-translation into English. This process preserved the intended meaning of all items while ensuring cultural appropriateness for the target population. Prior to enrollment, eligible individuals received a clear explanation of the study’s purpose, procedures, and confidentiality safeguards. We obtained written informed consent from each participant before their involvement. Trained research personnel supervised the data collection process, providing assistance and clarifying questions as participants completed the survey on electronic devices. The questionnaire captured a wide range of variables structured into multiple domains. Socio-demographic data included age, sex, marital status, education, employment, and insurance coverage. Lifestyle and behavioral factors, including physical activity, tobacco use, and dietary habits, were assessed. All comorbidities—such as obesity, hypertension, hypercholesterolemia, diabetes, and heart disease—were based on self-reported, physician-diagnosed history. Specifically, participants were asked if a healthcare provider had ever informed them of these conditions. We did not perform direct clinical measurements, laboratory testing, or medical record verification. Similarly, the primary outcomes were based on the lifetime history of an HIV or gonorrhea diagnosis, as reported by the participant. These self-reported histories served as the primary outcome variables. Of the 14,239 individuals invited to participate, all 14,239 completed the survey, representing a 100% response rate. We attribute this high completion rate to the user-friendly digital interface and our recruitment strategy. Trained coordinators provided on-site assistance at each PHC, ensuring participants could successfully navigate and submit the electronic questionnaire.

### 2.5. Pilot Testing and Instrument Validation

Prior to implementation, the electronic survey was pilot-tested in the Hail region. This site was chosen because it is geographically and administratively distinct from the primary study location. One hundred individuals participated in this phase to evaluate the clarity, content coverage, and usability of the questionnaire. During testing, participants provided feedback on their understanding of routine health screening questions, such as those regarding blood pressure and cholesterol. Following the pilot, the research team adjusted survey items for better precision and readability. To further enhance cultural relevance, we conducted a focus group with 20 key informants, including healthcare professionals and community representatives. Their insights informed final refinements to the wording and structure. To establish test–retest reliability, the original 100 pilot participants completed the revised tool a second time after a short interval.

The analysis yielded a test–retest reliability coefficient of 0.83, indicating high consistency over time. Similarly, internal consistency was confirmed with a Cronbach’s alpha of 0.83, demonstrating robust reliability within the current study population. We culturally adapted the questionnaire into Arabic using a rigorous forward-back translation process to maintain conceptual equivalence. To ensure comparability with international data, we maintained scoring procedures for comorbidities in accordance with original validation studies. A panel of public health experts, epidemiologists, and clinicians reviewed the questionnaire to ensure all items adequately captured the domains relevant to STI risk. Furthermore, face validity was affirmed by both the research team and 20 local informants, who confirmed the instrument was appropriate for the local context. Finally, we evaluated conceptual alignment by verifying that related items—such as lifestyle factors and chronic disease history—demonstrated expected relationships based on established epidemiological evidence. These reviews confirmed that the tool was fit for the specific objectives of this cross-sectional survey.

### 2.6. Data Analysis Approach

Analysis began with descriptive statistics to characterize the study population. We reported frequencies and percentages for categorical variables—including gender, marital status, employment, and the presence of chronic conditions—to provide a detailed overview of the sample. Prevalence estimates for self-reported HIV and gonorrhea were calculated with corresponding 95% confidence intervals (CIs) using standard binomial approximation methods. To examine preliminary associations, we conducted univariate logistic regression analyses for both primary outcomes. For each potential associate, we calculated odds ratios (ORs) and 95% CIs. During this initial screening, a $p$-value threshold of 0.25 was used to identify variables for inclusion in the final models. Subsequently, we developed multivariable logistic regression models to isolate the independent determinants of each outcome among Saudi participants. The modeling strategy utilized a conceptual framework where covariates were selected based on both statistical relevance in univariate analysis (*p* < 0.25) and theoretical significance established in prior epidemiological literature [24]. This threshold was chosen to ensure that potential confounders were not prematurely excluded. Due to the distinct nature of the included variables—which span unique demographic, behavioral, and clinical domains—and the exceptionally large sample size (*n* = 14,239), the model maintains high statistical stability. This large sample size ensures narrow standard errors and minimizes the risk of unstable coefficients typically associated with multicollinearity. Model fit was assessed using the Hosmer-Lemeshow goodness-of-fit test, with non-significant results (*p* > 0.05) indicating that the models appropriately fit the data. All statistical analyses were performed using IBM SPSS Statistics, version 26.

## 3. Study Results

### 3.1. Demographic and Health-Related Characteristics of the Study Population

Table 1 summarizes the demographic and health-related characteristics of the 14,239 participants. The cohort was predominantly aged ≥ 50 years (66.0%), married (65.3%), and lacked health insurance (75.7%). Over half of the participants (51.5%) had attained a college or university education. Regarding lifestyle and health status, 27.7% were smokers, 60.7% engaged in regular exercise, and 5.2% were classified as obese. Clinical comorbidities included diabetes mellitus (12.4%) and hypertension (11.1%). The self-reported lifetime prevalence was 2.6% (95% CI: 2.35–2.87%) for HIV and 3.1% (95% CI: 2.83–3.40%) for gonorrhea.

### 3.2. Sociodemographic Predictors of Sexually Transmitted Diseases

#### 3.2.1. Findings of Univariate Analysis

Univariable analysis was conducted using a *p* < 0.25 threshold to identify potential predictors for multivariable modeling (Table 2). For both HIV and gonorrhea, age (<50 years), female sex, and possession of insurance coverage were significantly associated with higher odds of infection (*p* < 0.05). Higher educational attainment was significantly associated with HIV (OR = 1.37, 95% CI: 1.08–1.73) but not with gonorrhea. Marital status and employment did not reach statistical significance at the *p* < 0.05 level during this initial screening phase.

#### 3.2.2. Findings of Multivariable Analysis

The multivariable logistic regression analysis identified several independent predictors for both outcomes (Table 3). After adjusting for confounders, age under 50 years remained a strong predictor for HIV (AOR = 2.19, 95% CI: 1.72–2.81) and gonorrhea (AOR = 1.57, 95% CI: 1.24–1.99). Females and married individuals consistently exhibited higher adjusted odds for both infections. Notably, insurance coverage remained significantly associated with higher odds for both HIV (AOR = 2.01, 95% CI: 1.63–2.50) and gonorrhea (AOR = 1.88, 95% CI: 1.54–2.29). Detailed AORs and confidence intervals for all sociodemographic variables are provided in Table 3.

### 3.3. Behavioral Risk Factors and Co-Morbidities Associated with Sexually Transmitted Diseases

#### 3.3.1. Findings of Univariate Analysis

Univariable analysis of behavioral and clinical factors revealed statistically significant associations (*p* < 0.001) for all examined variables across both HIV and gonorrhea (Table 4). Smoking was associated with a markedly increased likelihood of infection, with odds ratios of 8.39 (95% CI: 6.62–10.64) for HIV and 5.30 (95% CI: 4.34–6.47) for gonorrhea. Furthermore, all assessed metabolic and clinical comorbidities exhibited exceptionally strong associations in this unadjusted analysis. Notably, heart disease and obesity demonstrated the highest magnitudes of association, with odds ratios exceeding 30.0 for both outcomes. Hypertension and hypercholesterolemia also showed strong positive associations with self-reported infections. These clinical variables were subsequently included in the multivariable modeling to evaluate their independent predictive value after adjusting for demographic confounders.

#### 3.3.2. Findings of Multivariable Analysis

The multivariable logistic regression analysis identified several independent behavioral and clinical predictors for both outcomes (Table 5). Smoking remained a strong determinant for both HIV (AOR = 4.79, 95% CI: 3.44–6.69) and gonorrhea (AOR = 2.41, 95% CI: 1.85–3.14). Among clinical comorbidities, obesity (HIV: AOR = 11.55, 95% CI: 8.59–15.53; Gonorrhea: AOR = 9.02, 95% CI: 6.94–11.73) and heart disease (HIV: AOR = 11.31, 95% CI: 8.20–15.59; Gonorrhea: AOR = 8.77, 95% CI: 6.55–11.74) exhibited the strongest independent associations. Hypertension and hypercholesterolemia also remained important predictors (*p* < 0.05) for both infections. Notably, diabetes mellitus did not reach statistical significance as an independent predictor in either model (*p* > 0.05).

## 4. Discussion

This study provides novel insights into the prevalence and determinants of HIV (2.6%) and gonorrhea (3.1%) among primary healthcare attendees in Saudi Arabia. These estimates underscore a continuing public health challenge in a context traditionally considered to have lower STI burdens. However, as these figures are based on self-reported physician diagnoses without laboratory confirmation, they may be subject to underreporting, recall bias, or social desirability effects.

The 2.6% HIV prevalence is higher than previously reported in Saudi Arabia [22], potentially reflecting prior underreporting, limited routine screening, or increased population mobility. Globally, while sub-Saharan Africa bears the highest burden [25,26]. However, Middle Eastern countries have traditionally reported low prevalence due to cultural, religious, and behavioral factors [27,28]. The finding of a 3.1% prevalence of gonorrhea similarly calls attention to the silent spread of bacterial STIs. These infections often remain underdiagnosed due to a high frequency of asymptomatic cases and persistent social stigma. These estimates are broadly consistent with emerging evidence from other Middle Eastern and North African (MENA) countries, which show rising STI cases attributed to urbanization, changing sexual behaviors, and increased access to healthcare facilities facilitating case detection [14,15].

Our gonorrhea estimate (3.1%) is higher than the pooled regional average of 1.9% but lower than rates seen in high-risk clinical populations [13]. Similarly, systematic reviews covering over 20 million participants across 23 MENA countries highlight a substantial yet underrecognized STI burden. Most countries in this region still lack national screening programs and rely primarily on data from symptomatic populations [14]. Longitudinal analyses from the Global Burden of Disease Study indicate that age-standardized STI rates in the region have declined modestly. However, the absolute number of cases more than doubled between 1990 and 2021, reaching 55.8 million, due to demographic growth and persistent gaps in prevention and early detection [15]. These comparisons suggest that the prevalence observed in our study may reflect both increased detection among healthcare attendees and a genuine burden within the population.

The multivariable analysis revealed several independent determinants for HIV and gonorrhea. Key factors included demographic characteristics—such as age, sex, and marital status—alongside clinical and behavioral indicators, including smoking, hypertension, obesity, hypercholesterolemia, and heart disease. Given the cross-sectional nature of this study, these relationships are reported as statistical associations. We cannot determine a temporal sequence or causal link between these factors and the onset of infection. Some associations, such as the elevated odds among individuals with higher education, may appear counterintuitive. This could reflect increased mobility, broader social networks, and engagement in behaviors that confer higher exposure risk despite greater knowledge of preventive measures. Alternatively, it may reflect residual confounding or social desirability bias in self-reporting of risk behaviors.

Younger age (<50 years) was a key factor, consistent with global patterns where reproductive-aged adults engage in higher-risk behaviors [29,30]. This reinforces the need for youth-focused prevention and targeted screening. Furthermore, the finding that females were at higher risk highlights biological susceptibility and socio-cultural vulnerabilities, including barriers to confidential services [31,32]. Addressing these gaps requires culturally sensitive interventions and destigmatization efforts.

The association with higher education may appear counterintuitive but aligns with evidence linking education to increased mobility and diverse sexual networks [31,33,34,35]. In conservative contexts, higher-educated individuals may engage in more discreet behaviors [36,37], highlighting the need for tailored messaging. Similarly, the nearly two-fold increase in odds among married individuals suggests that marriage does not guarantee protection against infection. This may be due to factors such as extramarital relations or a lack of consistent condom use [38,39].

The link between health insurance and higher infection odds likely reflects “detection bias,” where insured individuals have greater access to testing [40,41,42,43]. Similarly, smoking showed a strong association with both infections, potentially serving as a marker for high-risk lifestyle clusters. Conversely, variables such as diabetes lost statistical significance after multivariable adjustment. This indicates that initial associations were likely confounded by age or cardiovascular comorbidities, highlighting the necessity of multivariable modeling to prevent the overestimation of effects.

Chronic health conditions, including hypertension, obesity, hypercholesterolemia, and heart disease, were independently linked to higher odds of both infections. Obesity and heart disease, in particular, showed very strong associations, with adjusted odds ratios exceeding nine and eleven times, respectively, for gonorrhea and HIV. The exceptionally high AORs observed for these clinical comorbidities necessitate a nuanced interpretation. These magnitudes likely reflect a combination of detection bias and healthcare utilization bias, as individuals with chronic conditions frequently interact with the healthcare system, increasing their probability of being screened for or informed of other diagnoses, including STIs. Furthermore, the possibility of reverse causality cannot be excluded in a cross-sectional design; for instance, the management of a known infection may lead to the subsequent identification of metabolic comorbidities. Finally, despite adjusting for several sociodemographic factors, residual confounding from unmeasured variables—such as specific high-risk sexual behaviors or deeper clinical histories—may further inflate these associations. Consequently, these values should be viewed as indicators of strong clinical clustering within a healthcare-seeking population rather than purely independent biological risks. While these relationships may initially seem surprising, emerging evidence suggests that metabolic disorders can impact immune function and systemic inflammation. These physiological changes may, in turn, increase an individual’s vulnerability to infections [44,45,46]. Moreover, these conditions may reflect underlying socioeconomic and lifestyle factors such as poor diet, physical inactivity, and limited healthcare access, which also contribute to STI risk [47,48]. Individuals with chronic diseases often have more frequent healthcare interactions, which increases their opportunities for STI screening and diagnosis. Consequently, this may lead to a higher observed prevalence due to detection bias. These findings underscore the importance of integrated care models that address both infectious and non-communicable diseases holistically.

Variables such as diabetes and education lost statistical significance after multivariable adjustment. This shift suggests that their apparent associations in unadjusted analyses were likely confounded by factors such as age, obesity, and cardiovascular comorbidities. The attenuation of these effects indicates that diabetes and education may not serve as independent determinants; instead, they likely act through shared pathways with more direct risk factors. Consequently, these findings highlight the value of multivariable modeling in isolating robust predictors and preventing the overestimation of effects. We have incorporated these clarifications to improve the transparency and interpretation of our results.

While the statistical significance of these determinants is clear, their clinical relevance must be interpreted with caution. The high adjusted odds ratios for metabolic conditions and smoking may partially reflect the characteristics of a healthcare-seeking population rather than absolute risk in the general community. Clinically, these findings suggest that primary care providers should consider metabolic and lifestyle risk profiles as indicators for STI screening, even in traditionally low-risk populations. However, establishing definitive clinical guidelines requires moving beyond these observed associations. Future longitudinal research is essential to this effort. Prospective studies that track participants over time and utilize laboratory-confirmed diagnoses will be critical to validating these findings. Such research is necessary to determine the true temporal and causal pathways between metabolic health and STI acquisition.

### Strengths and Limitations

This study possesses several notable strengths. First, the large sample size of over 14,000 participants enhances the statistical power and precision of the prevalence estimates. This scale also facilitates a robust analysis of independent determinants for each infection. Our use of a multi-stage stratified random sampling technique across diverse urban and suburban populations in Riyadh increases the representativeness of these findings; consequently, generalizability within similar Saudi Arabian contexts is improved. Furthermore, the comprehensive data collection instrument—incorporating socio-demographic, behavioral, and clinical factors—allowed for a holistic evaluation of risk. Finally, the rigorous pilot testing and validation process ensured the reliability and cultural relevance of the questionnaire.

This study has several limitations that warrant consideration. First, the cross-sectional design precludes causal inference between the identified determinants and infection outcomes. Second, the study population was restricted to individuals attending primary healthcare centers. This introduces potential selection bias, as healthcare-seeking individuals may exhibit different behaviors than the general population, potentially limiting the generalizability of these findings to the broader Saudi population. A critical measurement limitation is the reliance on self-reported, physician-diagnosed history for STIs and comorbidities. Because we lacked laboratory confirmation or medical record verification, the data are susceptible to social desirability bias and underreporting. This reliance on participant awareness likely explains the unexpectedly low obesity prevalence (5.2%) compared to national trends. Such a discrepancy suggests that our data primarily captures individuals who are aware of their clinical status, rather than the true physiological prevalence. Additionally, the sensitive cultural and legal context made it unfeasible to collect biological specimens or detailed data on sexual behaviors and partner characteristics. The absence of these variables restricts the depth of the behavioral risk assessment. Finally, as this study was limited to a single region, the applicability of these results to other geographical areas remains to be established. Future prospective research utilizing laboratory-confirmed diagnoses is essential to validate these findings.

## 5. Conclusions and Policy Implications

This study provides preliminary evidence that the burden of HIV and gonorrhea among primary healthcare attendees in Saudi Arabia is linked to demographic, behavioral, and metabolic factors. Due to the cross-sectional design and the reliance on self-reported diagnoses, causal inferences cannot be established. While the findings identify potential high-risk groups, including younger individuals, women, smokers, and those with metabolic comorbidities, these associations may be influenced by detection bias or residual confounding. Future longitudinal studies incorporating laboratory-confirmed diagnoses and detailed behavioral data are essential to validate these findings and clarify causal pathways. Such data will be critical for informing culturally sensitive STI prevention and control strategies within Saudi Arabia and the broader MENA region.

Overall, this study identifies important associations between demographic, metabolic, and behavioral factors and self-reported HIV and gonorrhea. While these findings provide a valuable epidemiological baseline for Saudi Arabia, the magnitude of the associations underscores the need for a balanced interpretation that prioritizes clinical context over statistical values alone. Strengthening integrated surveillance systems and transitioning toward longitudinal research frameworks will be vital for developing evidence-based prevention and screening strategies in the region.

## Figures and Tables

**Table 1:** Demographic and Health-Related Characteristics of the Study Population (*n* = 14,239).

Characteristic	Category	*n*	%
Age (Years)	<50 years	4848	34.0
	≥50 years	9391	66.0
Marital Status	Married	9300	65.3
	Single	4939	34.7
Employment	Employed	7317	51.4
	Unemployed	6922	48.6
Insurance coverage	No	10,782	75.7
	Yes	3457	24.3
Education Status	Less than college education	4509	31.7
	At least college education	9730	68.3
Smoking	No	10,297	72.3
	Yes	3942	27.7
Obesity	No	13,502	94.8
	Yes	737	5.2
Diabetes Mellitus	No	12,474	87.6
	Yes	1765	12.4
High Blood Pressure	No	12,659	88.9
	Yes	1580	11.1
HIV AIDS	No	13,866	97.4
	Yes	373	2.6
Gonorrhea	No	13,797	96.9
	Yes	442	3.1

**Table 2:** Sociodemographic Predictors of Sexually Transmitted Diseases among Saudis at primary healthcare settings in Riyadh (*n* = 14,239): Findings of Univariate analysis.

Predictors	HIV				Gonorrhea			
		95% CI		*p*-Value		95% CI		*p*-Value
	OR	LL	UL		OR	LL	UL	
Age								
At least 50 years	1.00			<0.001	1.00			0.01
<50 years	1.63	1.32	2.00		1.29	1.07	1.57	
Sex								
Male	1.00			<0.001				<0.001
Female	1.77	1.41	2.21		1.64	1.34	2.01	
Education								
Less than college education	1.00			0.01	1.00			0.30
At least college education	1.37	1.08	1.73		1.12	0.91	1.37	
Marital status								
Single	1.00			0.25	1.00			0.21
Married	1.14	0.91	1.42		1.14	0.93	1.39	
Employment status								
Unemployed	1.00			0.81	1.00			0.28
Employed	1.03	0.84	1.26		0.90	0.75	1.09	
Insurance coverage								
No	1.00			<0.001	1.00			<0.001
Yes	2.00	1.62	2.48		1.84	1.51	2.25	

OR: Odds ratio; 95% CI: 95% confidence intervals.

**Table 3:** Sociodemographic Predictors of Sexually Transmitted Diseases among Saudis at primary healthcare settings in Riyadh: Multivariate analysis.

Predictors	HIV				Gonorrhea			
		95% CI		*p*-Value		95% CI		*p*-Value
	AOR	LL	UL		AOR	LL	UL	
Age								
At least 50 years	1.00			<0.001	1.00			<0.001
<50 years	2.19	1.72	2.81		1.57	1.24	1.99	
Sex								
Male	1.00			<0.001	1.00			<0.001
Female	1.67	1.33	2.09		1.59	1.30	1.96	
Education								
Less than college education	1.00			0.04	NA			
At least college education	1.29	1.02	1.64					
Marital status								
Single	1.00			<0.001	1.00			0.002
Married	1.76	1.35	2.30		1.49	1.16	1.91	
Insurance coverage								
No	1.00			<0.001	1.00			<0.001
Yes	2.01	1.63	2.50		1.88	1.54	2.29	

AOR: Adjusted OR calculated in the multivariable analysis after controlling for mutual adjustment with other predictors that were significantly associated with HIV and Gonorrhea in Univariate Analysis; NA: Not applicable because the variable did not meet the *p*-value threshold of <0.25 at univariate analysis; Insurance: Having any form of health insurance coverage at the time of survey completion.

**Table 4:** Behavioral risk factors and co-morbidities associated with Sexually Transmitted Diseases in Saudi Arabia: Univariate analysis.

Predictors	HIV				Gonorrhea			
		95% CI		*p*-Value		95% CI		*p*-Value
	OR	LL	UL		OR	LL	UL	
Smoking								
No	1.00			<0.001				<0.001
Yes	8.39	6.62	10.64		5.30	4.34	6.47	
Diabetes								
No	1.00			<0.001	1.00			<0.001
Yes	4.81	3.88	5.96		4.40	3.60	5.38	
Hypertension								
No	1.00			<0.001	1.00			<0.001
Yes	12.32	9.96	15.23		8.63	7.10	10.48	
Obesity								
No	1.00			0.001	1.00			<0.001
Yes	42.95	34.23	53.90		31.44	25.55	38.69	
Hypercholesterolemia								
No	1.00			<0.001	1.00			<0.001
Yes	19.74	15.84	24.59		15.43	12.66	18.82	
Heart disease								
No	1.00			<0.001	1.00			<0.001
Yes	67.69	53.45	85.73		45.77	36.90	56.77	

OR: Odds ratio OR: Odds ratio; 95% CI: 95% confidence intervals; Obesity: Defined as body mass index (BMI) ≥30 kg/m^2^, based on self-reported height and weight; Hypertension: Self-reported history of physician-diagnosed hypertension or current use of antihypertensive medications; Diabetes: Self-reported history of physician-diagnosed diabetes or current use of antidiabetic medications; Heart Disease: Self-reported history of physician-diagnosed cardiovascular disease, including ischemic heart disease, heart failure, or prior myocardial infarction; Hypercholesterolemia: Self-reported history of physician-diagnosed high cholesterol or current use of lipid-lowering therapy; Smoking: Current use of any tobacco products (cigarettes, shisha, or smokeless tobacco).

**Table 5:** Behavioral risk factors and co-morbidities associated with Sexually Transmitted Diseases in Saudi Arabia: Multivariate analysis.

Predictors	HIV				Gonorrhea			
		95% CI		*p*-Value		95% CI		*p*-Value
	AOR	LL	UL		AOR	LL	UL	
Smoking								
No	1.00			<0.001				<0.001
Yes	4.79	3.44	6.69		2.41	1.85	3.14	
Diabetes								
No	1.00			0.68	1.00			0.30
Yes	1.08	0.75	1.57		1.19	0.86	1.64	
Hypertension								
No	1.00			<0.001	1.00			0.02
Yes	2.58	1.79	3.72		1.49	1.07	2.08	
Obesity								
No	1.00			0.001	1.00			0.001
Yes	11.55	8.59	15.53		9.02	6.94	11.73	
Hypercholesterolemia								
No	1.00			<0.001	1.00			<0.001
Yes	2.24	1.57	3.20		2.53	1.85	3.45	
Heart disease								
No	1.00			<0.001	1.00			<0.001
Yes	11.31	8.20	15.59		8.77	6.55	11.74	

AOR: Adjusted OR calculated in the multivariable analysis after controlling for mutual adjustment with other predictors that were significantly associated with HIV and Gonorrhea in Univariate Analysis.

## Data Availability

Data supporting this article is the property of King Fahad Medical City and cannot be available freely; however, we have added all related data within the article.

## Abbreviations

OR	Odds ratio
AOR	Adjusted odds ratio
95% CI	95% confidence intervals
LL	Lower limit
UL	Upper limit
NA	Not applicable
